# Where Is the ‘Oma’ in Multiple Myeloma? Origins and Limitations of some Myeloma‐Related Terminology

**DOI:** 10.1002/jha2.70326

**Published:** 2026-06-06

**Authors:** Jecko Thachil, David P. Steensma

**Affiliations:** ^1^ Department of Haematology Manchester University Hospitals Manchester UK; ^2^ Ajax Therapeutics Cambridge Massachusetts USA

**Keywords:** immunoglobulin, myeloma, paraprotein

## Abstract

**Background:**

Multiple myeloma, often simply called ‘myeloma’, is a clonal plasma cell malignancy that constitutes about one‐tenth of all cancers managed by haematologists. Although the disease was first clearly described in the first half of the 19th century, it was only called multiple myeloma several decades later.

**Aims:**

This review summarizes myeloma‐related terminology

**Content:**

Several terms are commonly used in myeloma clinics, including paraprotein, immunoglobulins, kappa and lambda chains and plasma cells; other terms, such as dysproteinemia, are now less commonly used.

**Summary:**

In this review, we look at the origins and limitations of these different myeloma‐related terminologies.

**Trial Registration:**

The authors confirm clinical trial registration is not needed for this submission

## 1 Introduction

The malignancy we now know as multiple myeloma (MM) has had a complex and interesting linguistic history, with the continuing use of potentially misleading terminology. Here, we discuss some of that history and the limitations of current terms.

## 2 The ‘oma’ in Myeloma

As with many other neoplasms, the term ‘myeloma’ ends with ‐oma, a suffix of Greek origin *(‐ωμα*) that indicates a process or action. According to the Oxford English Dictionary (OED), this Greek suffix was initially used in English words describing non‐medical topics, such as ‘diplomas’, which were once double‐folded (*diploun*) papers (‘oma’ here refers to the action of folding), or ‘scotoma’, a process of darkening that was later adopted as a neurology term [1]. But from the 19th century onward, words with ‑oma at the end were primarily used by pathologists to denote different types of tumours, particularly but not exclusively malignant neoplasms.

In this respect, MM should ideally stand for multiple tumours of the bone marrow. *Myelo‐* is a Greek prefix referring to bone marrow, or to the spinal cord, which was long ago mistaken for a peculiar form of marrow since it is surrounded by vertebral bodies; sometimes, this terminological similarity results in confusion with myeloid neoplasms, such as myelodysplastic syndromes. The first use of ‘multiple myeloma’ was by the obscure pathologist von Rusitzky in Kyiv in 1873 in a description of eight separate bone tumours thought to be arising from the marrow in a 47‐year‐old patient who presented with swelling of the temple, sternum and ribs [2, 3]. Von Rusitzky tried to distinguish myeloma from other bone malignancies by stating, ‘die mikroskopische wie makroskopische Beschaffenheit obiger Tumoren… den Namen “Myelome” zu geben’ (translated as, ‘the microscopic and macroscopic characteristics of the above tumours [justify us to classify them as a special class and] to give them the name “myelomas” [to denote the similarity of their structure to the bone marrow]’) [3].

## 3 Descriptions of Myeloma by Other Names

Myeloma as a distinct disease state was already known before it was baptized by von Rusitzky. In the 1840s, Macintyre in London encountered a patient, Thomas Alexander McBean, who presented with pathological fractures and oedema [4]. The patient's urine had stiffened his body linen despite the absence of urethral discharge and was of very high specific gravity [4]. McBean's urine was analysed by the best ‘chemical doctor’ in London, Henry Bence Jones, who found a precipitate in the urine with unique thermal properties and concluded that this protein in the urine was ‘hydrated deutoxide of albumen’ [2]. Bence Jones’ protein had no relation to albumen, but most proteins in the urine were called albumen in that era. Macintyre and Bence Jones exhorted clinicians to seek this ‘albumen oxide’ in all cases of what was then known as *mollities ossium*, a Latin term for soft bones similar to the currently used term osteomalacia and formerly used to describe many forms of bone disease [2]. (Patients had been described with pathological fractures resembling myelomatous bone disease before Macintyre's report, but without recognition of the proteinuria.) Thus, what would later be known as myeloma was initially a condition diagnosed based on Bence Jones proteinuria with accompanying bone changes.

In some countries, MM was given an eponym based on the physician who first described it or published an influential early paper. For example, ‘Kahler disease’ gained currency after an 1889 report of a physician with MM by the Vienna‐based pathologist Otto Kahler (1849–1893). In Italy, the term ‘Kahler‐Bozzolo disease’ recognized an 1897 paper by Torino‐based pathologist Camillo Bozzolo (1845–1920). The term ‘Kahler disease’ still maps to MM, C90.0, in the 10th edition of the International Classification of Diseases (ICD‐10) as well as in electronic medical records and databases based on ICD‐10 or early versions of the ICD.

## 4 The Para in Paraprotein

**FIGURE 1:**
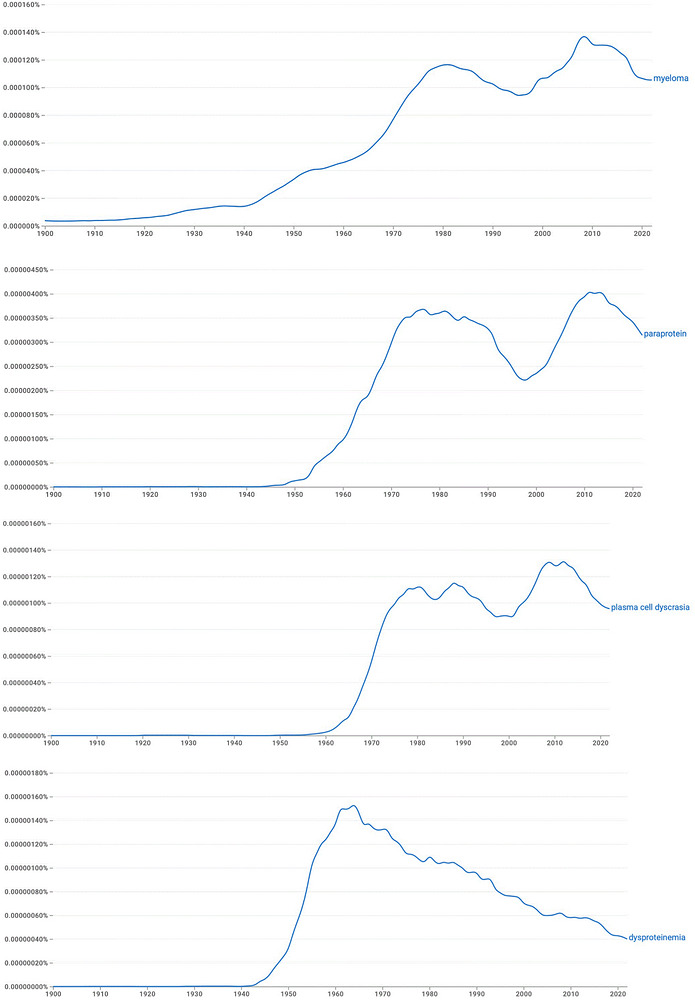
Google Ngram search (January 2026) on the incidence of use of the terms ‘paraprotein’, ‘dysproteinemia’ and ‘plasma cell dyscrasia’ in English‐language texts published since 1930. The use of ‘myeloma’ since the 19th century is shown for reference. The values on the *y*‐axis show what percentage of all words or phrases in the search term (i.e., the Ngram) in that year's corpus that match the search term. For example, a value of 0.000120% indicates that one in every 833,333 word pairs published in that year was the searched phrase. Note the differences in the scales in the *y*‐axis; myeloma has always been a more commonly used term than the other words.

MM is often said to be a ‘paraprotein’ disorder and sometimes a ‘dysproteinemia’. Where does the term ‘paraprotein’ originate? This expression was introduced in 1940 by the pathologist Apitz at Charité Hospital in Berlin, a few years before he died in an air raid [5]. In his article, ‘Protein metabolism disorders in plasmactyoma carriers’, Apitz stated, translated: ‘I have proposed summarizing them under the term “paraproteins” and understand by this to be a group of closely related, pathological protein products that occur in plasmacytoma, are easily degradable and readily crystallizable’ (i.e., *parallel* to other proteins) [5]. Swiss pathologist Roulet et al. considered that the use of the term ‘paraprotein’ was justified whenever an identified protein differed qualitatively from normal serum proteins [6]. PubMed and Google Ngram searches (Figure 1) show that use of ‘paraprotein’ has continued to climb since 1940, while ‘dysproteinemia’, a term introduced by the Swiss pathologist Ferdinand Wurhmann in 1945 and meant to emphasize the presence of abnormal protein in the blood, is falling out of favour.

## 5 Paraprotein to Immunoglobulins

Identification of paraproteins as monoclonal immunoglobulins involved three Swedish Nobel Prize Laureates, whose work mostly preceded the term ‘paraprotein’. First, Svante Arrhenius in Stockholm (Nobel Prize in Chemistry awarded in 1903) showed in the 1880s that salts, when dissolved, dissociated into positive and negative ions that enabled the solution to conduct an electric current [7]. Theodor Svedberg (prize awarded in 1926) continued the study of colloidal solutions in the Arrhenius laboratory and focused on developing the ultracentrifuge for the separation of proteins and the determination of their molecular weights. Finally, Tiselius (prize awarded in 1948), who began his career as an assistant in Svedberg's lab, showed that purified proteins migrated as homogenous bands in an electric field, as described in the next paragraph [7].

## 6 Alphabetical Jumble of Immunoglobulins

Tiselius’ work influenced the ‘alphabetical disorderliness’ of immunoglobulin nomenclature (G, M, A, D and E) that confounds many students today. He designed a *U*‐tube electrophoretic apparatus which could create sharp boundaries between protein and buffer solutions [8]. After testing horse serum using his apparatus, he found four distinct protein bands, albumin and three others, which he named ‘α,’ ‘β’ and ‘γ,’ and published this research in the *Transactions of the Faraday Society* [8]. Tiselius and Kabat in New York went on to demonstrate using electrophoresis that the gamma fraction of serum has the maximum amount of immunoglobulin, which was later called Ig**G** to denote gamma [9]. Tiselius's electrophoretic method, later simplified to use filter paper and widely adopted by clinical and research laboratories, had a huge impact on the detailed study of MM patients.

In 1944, Waldenström, who moved that year from Uppsala to Lund, described two patients who had an abnormal serum protein without bony lesions, whome he regarded as suffering from ‘myeloma without myeloma’. Waldenström initially wanted to include myeloma as a form of ‘thesaurismosis’, the term then used for storage diseases like Gaucher syndrome and derived from a Greek term for a storehouse or treasury [10]. In his publication, ‘Incipient myelomatosis or “essentia” hyperglobulinemia with fibrinognenopenia—a new syndrome?’ he described the presence of macroglobulins, now termed Ig**M** [10]. Waldenström's recognition of an abnormal protein in the gamma band in some apparently healthy people without any manifestations of myeloma or another disease, which he called ‘benign monoclonal gammopathy’ and other terms, has been known as ‘monoclonal gammopathy of undetermined significance’ (MGUS) since a seminal 1978 paper by Kyle at Mayo Clinic [11]. The term MGUS, as well as Kyle's later coinage ‘smouldering MM’, continues to be discussed and evolve, with a recent attempt to rename a subset of these disorders as ‘monoclonal gammopathy of clinical significance’ (MGCS) [12].

In the decade following Tiselius and Waldenström's work, a third immunoglobulin was discovered, which did not absorb out with either anti‐gamma or anti‐macroglobulin sera. The name of this β_2A_ globulin was truncated to α‐immunoglobulin (Ig**A**), although no globulins were ever identified in the α region of the electrophoretic peak [13, 14].

How the final two immunoglobulins became known as IgD and IgE in the 1960s was described by Pittsburgh immunologist Black in his detailed description of immunoglobulin nomenclature [14]. IgD's discoverers, immunologists Rowe and Fahey in Bethesda, wanted to christen this ‘IgB’ following the prior description of IgA, but this had been reserved for murine β‐globulin [14, 15]. They also couldn't choose IgC since C had no direct Greek equivalent, and the third letter of the Greek alphabet, gamma, was already taken, so they ended up using Ig**D** [15]. The ‘allergy immunoglobulin’, Ig**E**, was discovered by the couple Kimishige and Teruko Ishizaka, originally from Tokyo but then working at the Children's Asthma Research Institute and Hospital in Denver. The Ishizakas had been studying IgA antibody against blood group substances and found that IgA had no capacity to sensitize human skin [16]. They isolated the IgE from the serum of a person who was highly sensitive to ragweed pollen after they identified a globulin fraction that was neither IgG nor IgA. They called it serum factor E, which became IgE, where the ‘E’ in IgE stands for ‘erythema’ [17]. The World Health Organization developed the currently accepted nomenclature for immunoglobulins in 1964, recommending ‘an abbreviated notation to designate the major classes of immunoglobulins’ [18].

## 7 Kappa and Lambda Chains

In the 1950s, Korngold and Lipari in New York studied the antigenic relationship between Bence Jones proteins, myeloma‐associated abnormal serum globulins and normal gamma globulin using the Ouchterlony technique of immunodiffusion [19]. They concluded that Bence Jones proteins are produced by cells incapable of synthesizing the complete myeloma globulin, with the incompletely synthesized smaller proteins excreted into the urine [19]. In honour of these researchers, the two types of distinct proteins they examined were called κ and λ (Greek for K and L) after the first letters of Korngold's and Lipari's surnames; later, these were known as light chains once it was recognized that they were lower molecular weight components of normal immunoglobulin [4]

## 8 Why ‘Plasma’ Cells?

The monoclonal gamma globulins in MM are produced by clonal plasma cells. But why are plasma cells called ‘plasma’ cells? The etymology of plasma cell follows a circuitous journey commencing with the early 19th‐century concept of ‘protoplasm’.

Some early chemists believed that life originated with the right blending of chemical ingredients. In 1835, the French biologist and cytologist Félix Dujardin claimed to have succeeded in creating a living substance by crushing microscopic animals. The life substance Dujardin called ‘sarcode’ was later called ‘protoplasm’ (Greek ‘protos’ for ‘first’, and ‘plasma’ for ‘thing formed’) by the German botanist Hugo von Mohl. Protoplasm became the subject of intense research in the 19th century with the creation of the ‘Protoplasm Doctrine’: the concept that all living cells are made of protoplasm [20].

Waldeyer–Hartz in Berlin, perhaps best known for coining the term ‘neuron’, first mentioned plasma cells in his work ‘Über Bindegewebszellen’ (‘About Connective Tissue Cells’). He stated (translated), ‘In connective tissue, there occurs a group of cells that is almost as important, if not in number, then at least in distribution… I mean large, more rounded cells rich in protoplasm. Perhaps these cells may be provisionally given the name: “Embryonic cells of connective tissue” or simply “plasma cells”’ [21]. By ‘protoplasm,’ it is likely that he meant what is now known as the cellular contents, including the cytoplasm, nucleus and organelles. Waldeyer–Hartz's ‘plasma cells’, however, may actually have been mast cells; plasma cells as we now know them were first clearly described by the Spanish pathologist Santiago Ramón y Cajal [2]. Thus, ‘plasma cells’ clearly do not originate from the circulating plasma, but due to the 19th‐century belief that all cells originated from the protoplasm, that protoplasm could be visualized, and that these large cells found in connective tissue were particularly rich in protoplasm.

A localized neoplastic collection of plasma cells is called a ‘plasmacytoma’, a term first used by German pathologist Schridde in 1905 [22]. ‘Plasma cell dyscrasia’ is a newer term, although ‘dyscrasia’ from the Greek *κρᾶσις ‘*mixture’—indicating a bad combination—was used from the early 19th century to describe a range of diseases that it no longer applies to [23].

## 9 Conclusion and Looking Ahead

In the 21st century, MM is diagnosed when there is evidence of increased clonal plasma cells in combination with MM‐defining events, including organ damage and a high serum free light chain ratio. It is no longer a condition where diagnosis requires multiple tumours of the bone marrow, as it was in the 19th century. Apart from the subset of cases of plasmacytoma, myeloma is not really an ‘‐oma’ either, though patients presenting with advanced bony disease may have lytic lesions that contain collections of clonal plasma cells.

Once language gets established, it's hard to revise. For example, *influenza*, which we have known for many decades, is a viral illness and is not due to the ‘influence’ of the stars despite the coining of the term in 1504, yet millions of people still get an influenza vaccine every year. Likewise, *malaria* is not due to bad air (*mala aria*, dating to the Middle Ages), but those terms persist because they've taken on a life of their own, independent of an origin that reflected an incomplete view of pathology.

The immunoglobulin terminology, as irregular as it is, seems here to stay, but is it time to rename myeloma? If so, what would we call myeloma instead? Should we do away once and for all with the alternate terms like ‘plasma cell dyscrasia’ (dyscrasia = a disordered condition supposed to arise from a disproportionate mixture of the ‘humours’) and ‘dysproteinemia’? As shown in Figure 1, dysproteinemia seems likely to continue to slowly fade out naturally, but paraprotein and plasma cell dyscrasia persist.

## Funding

The authors have nothing to report.

## Ethics Statement

The authors have nothing to report.

## Consent

The authors have nothing to report.

## Conflicts of Interest

The authors declare no conflicts of interest.

## Data Availability

The authors have nothing to report.
